# Lesion viral burden, multidrug-resistant superinfection, and HIV-associated haematological vulnerability in hospitalised clade Ib mpox: a prospective cohort study in Uganda

**DOI:** 10.1016/j.ebiom.2026.106409

**Published:** 2026-08-05

**Authors:** Jennifer Serwanga, Raymond Ernest Kaweesa, Geoffrey Odoch, Brenda Nairuba, Deborah Mukisa, Rodney Abraham Tumusiime, Ruth Priscilla Nambi, Annie Daphine Ntabadde, Violet Ankunda, Tom Lutalo, Michael Selorm Avumegah, John Bosco Nsubuga, Christopher Nsereko, Pontiano Kaleebu

**Affiliations:** a Uganda Virus Research Institute (UVRI), Entebbe, Uganda; b MRC/UVRI & LSHTM Uganda Research Unit, Entebbe, Uganda; c London School of Hygiene & Tropical Medicine, Keppel Street, London, United Kingdom; d Entebbe Regional Referral Hospital, Entebbe, Uganda; e Coalition for Epidemic Preparedness Innovations (CEPI), Askekroken 11, 0277, Oslo, Norway

**Keywords:** Mpox, Clade Ib mpox, Lesion viral burden, Antimicrobial resistance, Bacterial superinfection, People living with HIV-1

## Abstract

**Background:**

Mpox has shifted to sustained human-to-human transmission across Africa, yet integrated triage incorporating viral burden, bacterial co-infection, antimicrobial resistance (AMR), HIV status, and routine biomarkers remain scarce.

**Methods:**

At Uganda's national mpox referral hospital, we prospectively enrolled 155 adults at 14 ± 2 days post-symptom onset; mpox was confirmed by lesion-swab qPCR (F3L), with clade assignment by whole-genome sequencing in a prespecified subset (March–April 2025). Lesion viral DNA burden was estimated using qPCR cycle threshold (Ct) values. Purulent lesions underwent EUCAST-standardised culture and susceptibility testing. Routine laboratory assessments included complete blood counts, C-reactive protein, serum chemistries, HIV serostatus, and plasma HIV-1 RNA.

**Findings:**

Median age was 30 years, and 73/155 (47%) had HIV infection. Multisite pain, particularly anogenital, was highly prevalent. Among 80 participants with purulent lesions selected for clinically suspected bacterial superinfection, all yielded bacterial growth; 35/80 (43.8%) were polymicrobial and predominantly multidrug-resistant Gram-negative bacilli. Susceptibility to first-line β-lactams and fluoroquinolones was low, whereas meropenem retained activity (51/61, 84%). Lesion viral burden did not differ by HIV serostatus and showed weak correlation with HIV-1 viraemia; higher burden was associated with leucocytosis, neutrophilia with left shift, elevated CRP, and hypoalbuminaemia. Genomes clustered within Clade Ib, without segregation by HIV status or clinical severity.

**Interpretation:**

Hospitalised adults with acute Clade Ib mpox in Uganda exhibited high lesion viral burden, frequent multidrug-resistant bacterial co-isolation, and an inflammatory haematological profile accentuated in participants living with HIV-1. These findings support consideration of integrating diagnostic microbiology, HIV viral load assessment, and antimicrobial stewardship into mpox case-management in endemic settings.

**Funding:**

This study was supported by the Coalition for Epidemic Preparedness Innovations (CEPI; Project ID PRJ-8284).

**Research in context**

**Evidence before this study**

We searched PubMed and major bibliographic databases up to March 2026 using the terms “mpox”, “monkeypox”, “viral load”, “cycle threshold”, “bacterial superinfection”, “antimicrobial resistance”, and “HIV”. Prior studies have described the evolving clinical phenotype of mpox, including mucocutaneous disease, severe pain, and frequent secondary bacterial complications, alongside evidence of sustained human-to-human transmission. A limited number of studies have examined viral load dynamics and systemic inflammatory responses, largely in non-African cohorts, while others have reported associations between advanced HIV infection and adverse clinical outcomes. Separately, antimicrobial resistance among bacterial pathogens in sub-Saharan Africa is well documented. However, these domains have been investigated independently. There remains a lack of integrated clinical datasets linking lesion-level viral burden with microbiological findings, antimicrobial susceptibility, and routinely available laboratory markers, particularly in African settings where Clade I (subclade Ib) mpox predominates. The relationship between HIV viraemia, lesion viral burden, and systemic inflammatory responses also remains incompletely defined.

**Added value of this study**

This prospective cohort study integrates lesion-level viral burden, microbiological findings, antimicrobial susceptibility patterns, and routine clinical laboratory markers in hospitalised adults with Clade Ib mpox in Uganda. We show that lesion viral burden, estimated using qPCR cycle threshold values, is not differentiated by HIV serostatus but is associated with a consistent inflammatory profile characterised by leucocytosis, neutrophilia, elevated C-reactive protein, and hypoalbuminaemia. We identify a high frequency of multidrug-resistant bacterial co-isolation among participants with purulent lesions, predominantly involving Gram-negative organisms with limited susceptibility to commonly used first-line antibiotics. In parallel, we demonstrate a distinct haematological profile among participants living with HIV, marked by anaemia, anisocytosis, and myeloid skewing, which is not captured by viral burden alone. By integrating virological, microbiological, and host-response data within a single analytic framework, this study provides clinically actionable insights relevant to inpatient management in resource-constrained settings.

**Implications of all the available evidence**

Taken together, these findings support a shift toward integrated, laboratory-informed clinical management of mpox in endemic and outbreak settings. Lesion viral burden appears to reflect systemic inflammatory activation rather than HIV status alone and may serve as a pragmatic biomarker for disease activity. The high prevalence of multidrug-resistant bacterial co-isolation underscores the need for culture-guided antimicrobial therapy and strengthened antimicrobial stewardship within mpox care pathways. The observed dissociation between HIV serostatus and lesion viral burden, alongside distinct haematological perturbations among participants living with HIV, highlights the importance of incorporating HIV viral load and routine laboratory markers into risk stratification. Embedding these integrated approaches into national case-management guidelines could improve patient outcomes, optimise antibiotic use, and strengthen epidemic preparedness in African health systems.

## Introduction

Mpox is a zoonotic infection caused by the Mpox virus,^1^, ^2^, ^3^ a large double-stranded DNA orthopoxvirus with well-described immune evasion capacity.^4^, ^5^, ^6^ Two major genetic clades are recognised: Clade I (Central Africa/Congo Basin) and Clade II (West Africa), which differ by approximately 0.5% at the genomic level, with Clade I historically associated with greater virulence.^4^^,^^7^^,^^8^ Clade I is further subdivided into sub-clades Ia and Ib.^9^, ^10^, ^11^ Since 2023, outbreaks across Africa have been driven predominantly by the emergent Clade Ib lineage,^12^, ^13^, ^14^ raising concern regarding its epidemic potential in densely connected social networks.

Concurrently, the clinical epidemiology of mpox has shifted from sporadic zoonotic spillover to sustained person-to-person transmission. Recent outbreaks increasingly feature genital and mucocutaneous disease, marked pain, lymphadenopathy, cellulitis, frequent secondary bacterial infections and prolonged recovery.^15^, ^16^, ^17^, ^18^ For health systems in endemic and newly affected regions, this evolving phenotype creates dual pressures: accurate assessment of viral burden and rational antimicrobial stewardship amid rising antimicrobial resistance (AMR).^19^^,^^20^

Although viral burden at the lesion is central to both transmission biology and tissue pathology, integrated datasets linking lesion-level viral burden with microbiology and routine inflammatory markers remain scarce. Prior studies have characterised the clinical spectrum of mpox and documented complications including pain, bacterial superinfection and ocular involvement.^15^^,^^21^^,^^22^ Case series in people living with HIV (PLHIV) report heterogeneous outcomes and delayed lesion resolution in some individuals,^23^ but the relationship between HIV replication and mpox lesion viral burden remains poorly defined. In parallel, high background rates of AMR among common skin and soft-tissue pathogens in sub-Saharan Africa complicate empiric therapy and highlight the importance of context-specific susceptibility data.^19^^,^^20^ Widely available biomarkers such as white blood cell differentials, C-reactive protein (CRP), and albumin are routinely used to stage inflammation and guide inpatient care,^24^^,^^25^ yet their relationship to mpox lesion viral burden has not been prospectively quantified in harmonised cohorts.

The field therefore lacks an integrated framework addressing three clinically relevant questions: whether lesion viral burden differs by HIV serostatus and viraemia; whether bacterial co-isolation and antimicrobial susceptibility patterns stratify lesion viral burden; and whether routine biomarkers exhibit a coherent inflammatory signature aligned with lesion viral load during acute disease. Each component has largely been studied in isolation.^26^, ^27^, ^28^, ^29^

We therefore tested the hypothesis that higher lesion viral burden (reflected by lower Ct values) co-occurs with bacterial co-isolation and inflammatory haematological activation, and that HIV viraemia, rather than serostatus alone, modifies these relationships. This study integrates lesion virology, microbiology, and routine laboratory markers in a hospitalised Ugandan mpox cohort to inform risk stratification in resource-constrained settings.

## Methods

Between 3 March and 10 April 2025, we conducted a prospective consecutive inpatient cohort study at Entebbe Regional Referral Hospital (ERRH), which served as Uganda's national mpox referral centre during the study period.^30^^,^^31^ Eligible adults admitted for mpox case management were consecutively enrolled. At admission, patients were categorised within the WHO admission triage classification^32^ as suspected, probable or confirmed mpox for triage purposes. These care-pathway classifications were not used for primary inferential analyses. The primary analytic cohort for this report comprised adults with retrospectively confirmed mpox by real-time quantitative PCR (qPCR) targeting the F3L gene; Clade Ib assignment was confirmed by whole-genome sequencing in a predefined subset, as previously described.^33^ Cycle threshold (Ct) values were used as an inverse proxy for lesion viral DNA burden.

At a harmonised acute timepoint (14 ± 2 days from symptom onset), we collected lesion swabs (skin, genital, and anal sites), whole blood, and saliva. For the primary Ct analysis, one lesion-linked qPCR result per participant was selected using a prespecified hierarchy, with genital swabs prioritised where available and alternative lesion-site swabs used otherwise, as detailed in the Supplementary Methods. Purulent lesions meeting predefined clinical criteria were sampled for aerobic bacterial culture and EUCAST-standardised susceptibility testing. HIV serostatus was determined using the national serial rapid-testing algorithm, and plasma HIV-1 RNA was quantified among PLHIV to objectively assess viral suppression. Routine complete blood counts and serum chemistries (including C-reactive protein and albumin) were measured using standardised laboratory procedures, relative to normal reference ranges.^34^^,^^35^ Whole-genome sequencing of Mpox virus was performed on a predefined subset of high-viral DNA specimens from enrolled participants to confirm clade assignment and characterise outbreak phylogeny.

Statistical analyses were conducted in R (v4.4.1). Continuous variables were summarised as medians with interquartile ranges and categorical variables as counts and percentages. Between-group comparisons were performed using the Wilcoxon rank-sum test, and associations between continuous variables were assessed using Spearman rank correlation. All tests were two-sided, and p < 0.05 was considered statistically significant. Missing data were not imputed; analyses used available data. Detailed laboratory protocols, assay parameters, sequencing workflows, and full analytical specifications are provided in the Supplementary Methods.

### Ethics

The study protocol was reviewed and approved by the Uganda Virus Research Institute Research and Ethics Committee (UVRI-REC; GC/127/1068) and the Uganda National Council for Science and Technology (UNCST; NS934ES). All participants provided written informed consent before enrolment and before collection of clinical data and biological specimens. Only adults were included in the analytic cohort reported in this manuscript. Participant data were de-identified before analysis, and all analyses were conducted in accordance with approved study procedures, national research governance requirements, and the ethical principles of the 2024 Declaration of Helsinki.

### Role of the funding source

This study was supported by the Coalition for Epidemic Preparedness Innovations (CEPI; Project ID PRJ-8284). The funder had no role in study design, participant enrolment, data collection, laboratory testing, data analysis, data interpretation, writing of the report, or the decision to submit the manuscript for publication. The corresponding author had full access to the study data and had final responsibility for the decision to submit for publication.

## Results

### Study population

A total of 157 patients were enrolled during the study period. Two paediatric participants were excluded, leaving 155 adults in the analytic cohort. Epidemiological interview data were available for 146/155 participants; two of these lacked an admission case-group label in the screening dataset, so descriptive analyses by admission classification used 144 participants. Laboratory analyses used all available data per assay: complete blood count and serum chemistry, n = 155; lesion bacterial culture, n = 80 participants with purulent lesions meeting prespecified criteria; HIV-1 RNA, n = 71/73 PLHIV; and genital lesion qPCR Ct analyses, n = 151 selected lesion-linked specimens from 155 participants (Table 1; Figure S1).

**Table 1:** Baseline demographic characteristics of the study population (n = 155).

Characteristic	Median (IQR)/n (%)	Female (col %)	Male (col %)
Total	155 (100)	81 (52.3)	74 (47.7)
Age (Years), Median (IQR)	30.0 (25.0, 35.0)	28.0 (24.0, 35.0)	30.5 (26.0, 35.0)
HIV Status			
HIV-Positive	73 (47.1)	46 (56.8)	27 (36.5)
HIV-Negative	82 (52.9)	35 (43.2)	47 (63.5)
HIV Viral load, median (IQR)^a^	52 (1, 27,800)	40 (1, 18,775)	1200 (62, 88,250)
HIV Viral load^a^ (copies per mL)^b^			
<200	39 (53.4)	30 (65.2)	9 (33.3)
200–<1000	4 (5.5)	1 (2.2)	3 (11.1)
1000–100,000	15 (21.1)	7 (15.2)	8 (29.6)
≥100,000	13 (17.8)	6 (13.0)	7 (25.9)
Not tested	2 (2.7)	2 (4.3)	0 (0.0)
Participants undergoing bacterial culture^c^			
Yes	80 (51.6)	48 (59.3)	32 (43.2)
No	75 (48.4)	33 (40.7)	42 (56.8)

Table 1 summarises the enrolled participant characteristics at admission (n = 155). The table summarises demographic and clinical features for 155 adults with mpox. Continuous data are shown as median (IQR); categorical data as n (%). HIV viral-load metrics are reported for participants living with HIV; thresholds are expressed in copies per mL (cp/mL), and “Not tested” denotes participants living with HIV-1 without an available viral-load measurement. “Organism isolated” indicates whether a pathogen was recovered on culture.

a Viral load for only HIV + participants.

b 71 of 73 had plasma HIV-1 RNA data.

c Participants undergoing bacterial culture” refers to those from whom purulent lesions were sampled for microbiological investigation.

### Self-reported exposures patterns suggest intimate and household contact

Structured exposure interviews were completed for 146 (94%) adults with retrospectively confirmed qPCR-positive mpox. Sexual intercourse (55/146, 37.7%), and direct skin-to-skin contact (53/146, 36.3%) were most reported, followed by contact with potentially contaminated clothing or bedding (34/146, 23.3%), mouth-to-skin contact (33/146, 22.6%), and prolonged face-to-face exposure (21/146, 14.4%). Exposure source was unknown in 45/146 (30.8%). Overall, self-reported patterns were consistent with predominant transmission through close intimate and household contact. Admission triage classifications are summarised in Table S1.

### Genital-predominant mpox independent of HIV strata

**Fig. 1:**
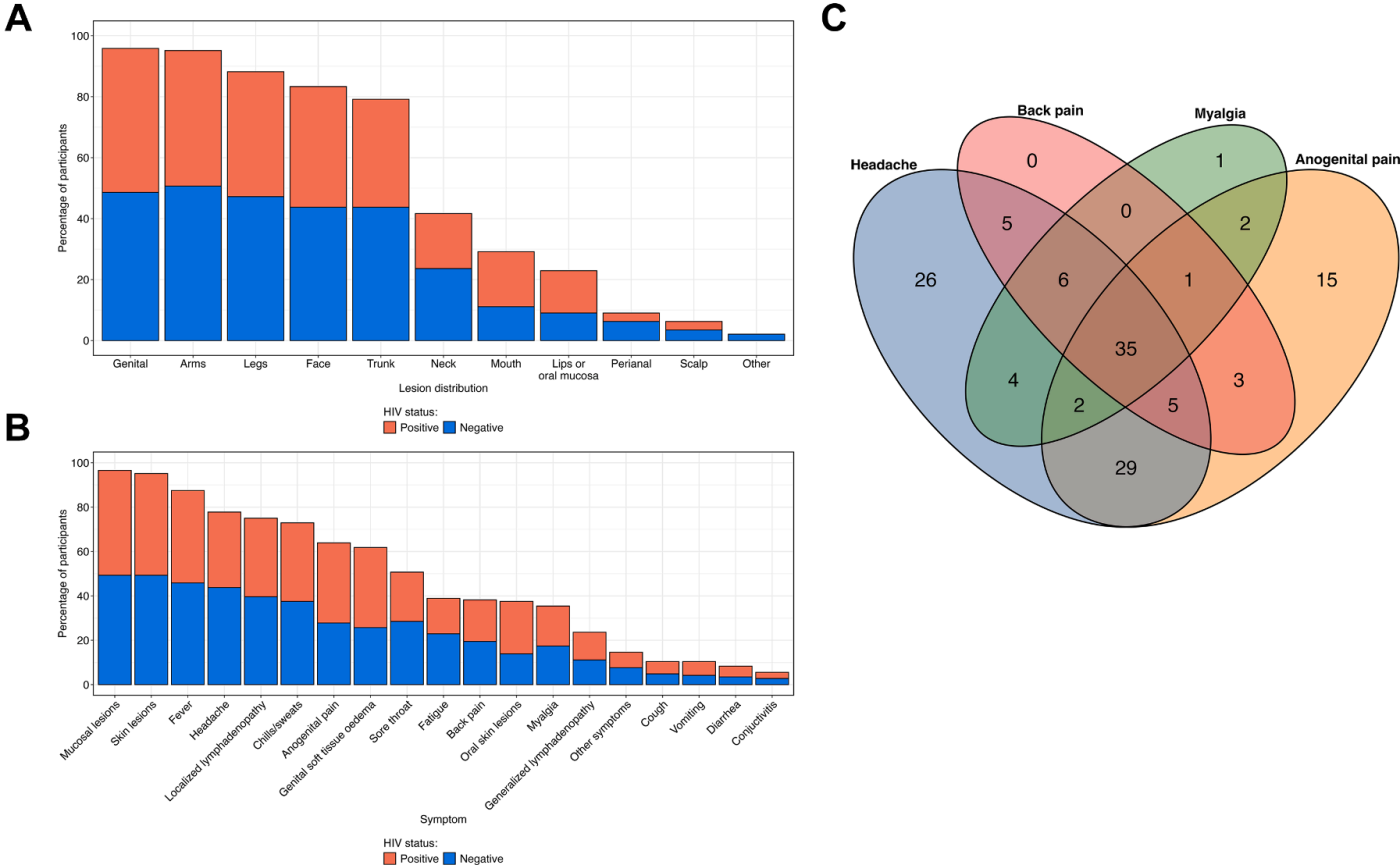
**Anatomical distribution of mpox rash, symptom prevalence and overlap of pain syndromes by HIV status**. **A**. Shows the anatomical distribution of mpox rash. Stacked bars represent the percentage of participants reporting lesions at each body site, stratified by HIV status (blue, participants without HIV-1; red, participants living with HIV-1). **B**. Summarises the symptom prevalence across the cohort with bars indicating the proportion of participants reporting each symptom, similarly, stratified by HIV status. **C**. Illustrates the overlap of four major pain syndromes, headache (112/134, 83·5%), anogenital pain (92/134, 68·7%), back pain (55/134, 41·0%), and myalgia (51/134, 38·1%), among participants reporting pain. Values within each segment indicate the number of individuals reporting each symptom alone or in combination. (A–B) n = 146 participants with available interview data; (C–D) n = 134 participants reporting pain.

Rash was the dominant clinical feature and was primarily genital in distribution, with frequent extension to the limbs, face, and trunk (each ≥∼80%; Fig. 1A). Involvement of the neck (∼40%) and oral cavity (∼30%) was less common, while perianal, scalp, and other sites were infrequent (≤10%).

Symptomatically, mucocutaneous lesions (∼90%) and fever (>80%) predominated, followed by headache, lymphadenopathy, and chills or sweats (each >70%; Fig. 1B). Pain burden was high: anogenital pain with associated genital soft-tissue oedema occurred in approximately two-thirds of participants. Sore throat (∼50%) and back pain or myalgia and fatigue (∼40% each) were reported less frequently. Respiratory, gastrointestinal symptoms (cough, vomiting, diarrhoea) and ocular symptoms were uncommon (each ≤10%), although rare severe ocular complications were documented (corneal ulcer and endophthalmitis). No meaningful differences were observed by HIV status in lesion distribution or symptom frequency, indicating a consistent genital-mucocutaneous phenotype across the cohort.

### Multisite pain was a dominant clinical feature of acute mpox

Pain was highly prevalent and anatomically multifocal. Among 146 participants assessed, 134 (91.7%) reported pain (Fig. 1C). Of 134 with resolved anatomical data, headache was most common (112/134; 83.5%), followed by anogenital pain (92/134; 68.7%), back pain (55/134; 41.0%), and myalgia (51/134; 38.1%). Co-localisation was frequent: 26.1% (35/134) reported concurrent headache, anogenital pain, back pain and myalgia. The most common dyadic overlap was headache with anogenital pain (29/134, 21.6%). Isolated symptoms were uncommon (headache alone 26/134; 19.4%; anogenital pain alone 15/134; 11.2%) (Fig. 1D). These data identify pain as a defining component of acute mpox morbidity.

### Multidrug-resistant bacterial co-isolation in mpox lesions

**Fig. 2:**
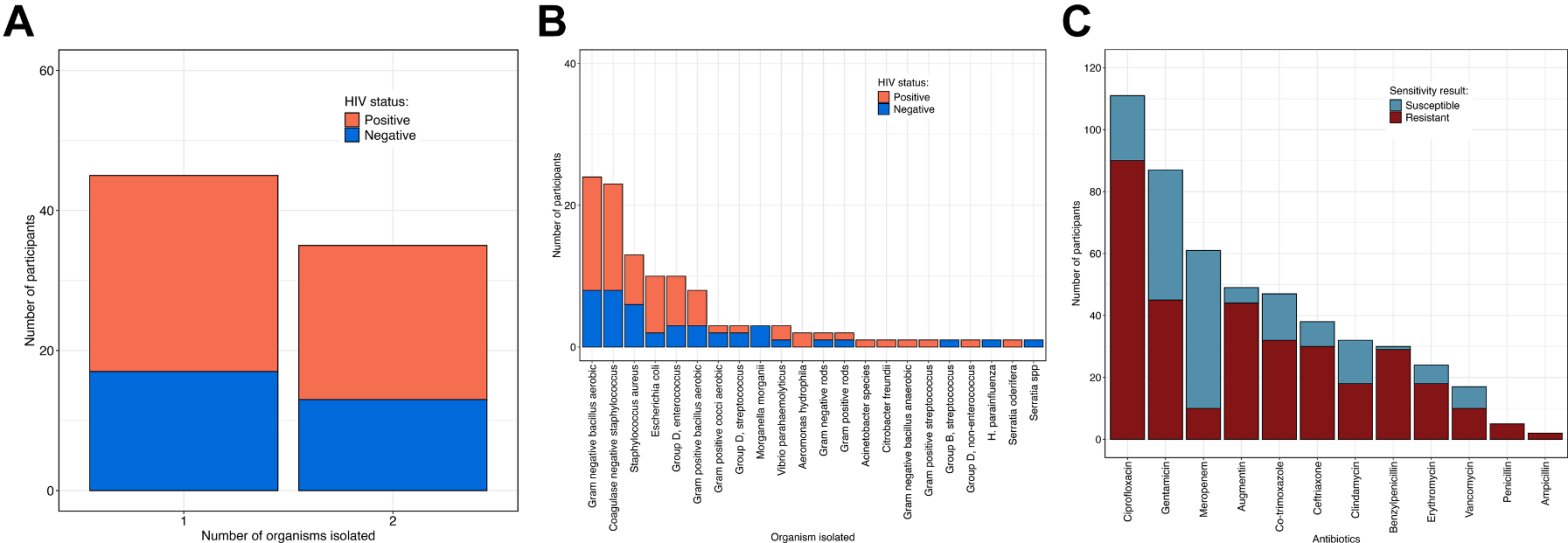
**Bacterial Isolates and Antimicrobial Susceptibility among hospitalised mpox patients**. Purulent lesion cultures were obtained from 80 of 155 hospitalised adults with mpox. **A**. Shows the number of bacterial organisms isolated per participant (one or two), stratified by HIV status (red, participants with HIV-1; blue, participants without HIV-1). **B**. Summarises the distribution of 13 bacterial taxa recovered from lesion cultures across 80 participants, with counts stratified by HIV status. Antimicrobial susceptibility profiles for isolates tested against a panel of 12 antibiotics (**C**); stacked bars exceed the number of cultured participants because individual isolates were tested against multiple antibiotics.

Among 155 hospitalised adults with mpox, purulent lesion cultures were obtained from 80 participants with clinically suspected bacterial superinfection, yielding bacterial growth that was classified into 13 distinct taxa (Fig. 2). Antimicrobial susceptibility testing against a panel of 12 antibiotics generated 503 organism-antibiotic combinations, of which 333 (66.2%) were interpreted as resistant and 170 (33.8%) as susceptible. Within the overall cohort, 73 of 155 participants (47.1%) were living with HIV and 82 (52.9%) were HIV-negative. Plasma HIV RNA measurements were available for 71 of 73 PLHIV (97.3%), with a median viral load of 52 copies per mL (IQR 1–27,800); 15 of 71 (21.1%) had viraemia ≥100,000 copies per mL (Fig. 2).

All 80 purulent lesion swabs yielded bacterial growth. Most participants had a single organism isolated (45/80, 56.3%), while polymicrobial infection involving two organisms was identified in 35 of 80 (43.8%). Participants undergoing culture were more frequently living with HIV (50/80, 62.5%) than those without HIV (30/80, 37.5%) (Fig. 2A). Across cultures, Gram-negative aerobic bacilli (24/80, 30.0%) and coagulase-negative staphylococci (23/80, 28.8%) were the most frequently recovered organisms. Less common isolates included Enterococcus spp., *Escherichia coli*, *Staphylococcus aureus*, and Group D streptococci, while *Citrobacter freundii* and *Acinetobacter* spp., were each identified in a single participant (Fig. 2B).

**Fig. 3:**
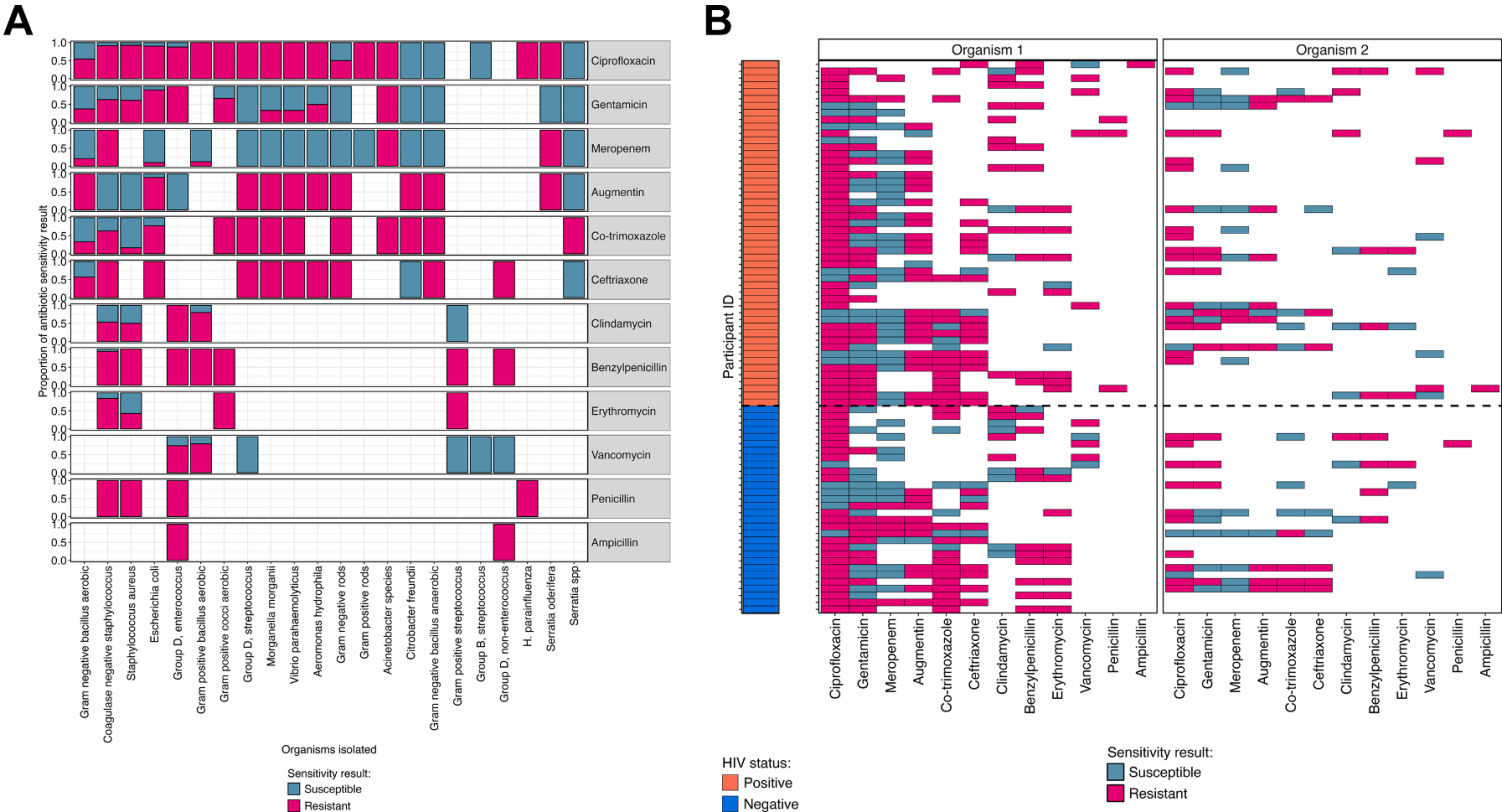
**Antibiotic susceptibility patterns among bacterial isolates from hospitalised mpox patients**. Among participants with bacterial cultures performed (n = 80), (**A**) summarises organism-specific antibiotic susceptibility profiles across tested antibiotics, showing the proportion of isolates interpreted as susceptible (teal) or resistant (magenta) for each organism-antibiotic combination. **B**. Displays participant-level susceptibility interpretations across antibiotics, stratified by HIV status (orange, participants with HIV-1; blue, participants without HIV-1), with results shown separately for Organism 1 and Organism 2, where two isolates were recovered from the participant. Not all antibiotics were tested against every isolate; denominators, therefore, vary by organism and drug, and susceptibility classifications follow routine clinical interpretation standards.

Antimicrobial susceptibility testing across the recovered isolates demonstrated widespread resistance to commonly used antibiotics (Figs. 2C and 3A, Table 2, Table S2). High resistance was observed for ciprofloxacin (90 resistant vs 21 susceptible isolates), amoxicillin–clavulanate (44 resistant vs 5 susceptible), ceftriaxone (30 resistant vs 8 susceptible), and co-trimoxazole (32 resistant vs 15 susceptible). Gentamicin demonstrated mixed activity (45 resistant vs 42 susceptible), whereas meropenem retained the greatest in-vitro activity (10 resistant vs 51 susceptible isolates).

**Table 2:** Antimicrobial Susceptibility Results of Bacterial Isolates Recovered from Cultured mpox lesions, Stratified by HIV Status.

Antibiotic	HIV status	Susceptible	Resistant	OR (95% CI)	p-value
Ciprofloxacin	Negative	8	34	1.01 (0.33, 2.96)	1
	Positive	13	56		
Gentamicin	Negative	18	15	1.49 (0.58, 3.93)	0.39
	Positive	24	30		
Meropenem	Negative	16	5	0.46 (0.09, 2.32)	0.29
	Positive	35	5		
Augmentin	Negative	2	13	1.57 (0.12, 15.50)	0.64
	Positive	3	31		
Co-trimoxazole	Negative	8	17	1.01 (0.25, 4.15)	1
	Positive	7	15		
Ceftriaxone	Negative	5	11	2.80 (0.44, 21.62)	0.24
	Positive	3	19		
Clindamycin	Negative	8	5	3.32 (0.64, 19.62)	0.15
	Positive	6	13		
Benzylpenicillin	Negative	1	14	2.09 (0.10, 133.48)	1
	Positive	0	15		
Erythromycin	Negative	2	9	0.51 (0.04, 4.72)	0.65
	Positive	4	9		
Vancomycin	Negative	3	3	1.69 (0.15, 20.06)	0.64
	Positive	4	7		
Penicillin	Negative	0	1	2.23 (0.02, 234.24)	1
	Positive	0	4		
Ampicillin	Negative	0	0		
	Positive	0	2		

Table 2 summarises antibiotic susceptibility results for bacterial isolates recovered from purulent lesions cultured from 80 participants (30 HIV-negative; 50 participants living with HIV-1). The table summarises 503 interpretable organism-antibiotic susceptibility results (198 from HIV-negative participants and 305 from participants living with HIV-1). For each antibiotic, counts represent the number of susceptible and resistant susceptibility interpretations recorded for all tested isolate–antibiotic combinations within each HIV stratum. Participants with multiple bacterial isolates could contribute more than one observation, and not all antibiotics were tested against every isolate; therefore, denominators vary by antibiotic. Odds ratios (ORs) with 95% confidence intervals compare susceptibility among isolate–antibiotic observations from participants living with HIV-1 vs those without HIV. All p values are two-sided.

When stratified by HIV status, 71 of 198 organism–antibiotic combinations (35.9%) derived from isolates recovered from participants without HIV were susceptible, compared with 99 of 305 (32.5%) among isolates from participants living with HIV-1. Overall susceptibility patterns were similar between the two groups, with resistance predominating across most antibiotics, while meropenem retained the greatest in vitro activity. Meropenem retained activity in both groups, with 10 susceptible vs four resistant results in HIV-negative participants and 30 susceptible vs four resistant results among participants living with HIV-1, whereas resistance predominated for most other antibiotics.

Participant-level heat map visualisation (Fig. 3B) illustrates the distribution of susceptibility interpretations across antibiotics for each participant and recovered organism, including individuals with multiple isolates. Across the antibiotic panel, resistant predominated across organism-antibiotic combinations, while meropenem susceptibility remained consistently preserved.

### HIV-associated anaemia, anisocytosis, and myeloid skewed CBC profiles in mpox

**Fig. 4:**
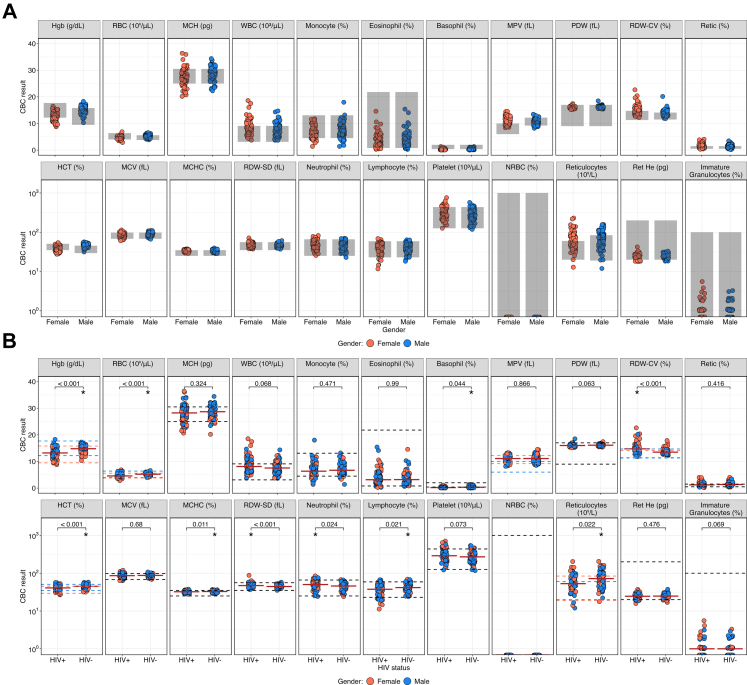
**Distribution of Complete Blood Count Parameters by Sex and HIV Status**. The figure summarises distributions of haematological parameters measured by complete blood count (CBC) across the study cohort (n = 155). Values are stratified by sex (red, female, blue, male), with grey bands indicating sex-specific reference ranges (**A**). Values are also stratified by HIV status (participants with HIV-1 vs participants without HIV-1), with dashed lines indicating laboratory reference limits (**B**). Each point represents an individual participant.

Complete blood count (CBC) analyses across the cohort (n = 155) revealed that most parameters clustered within sex-specific reference ranges (grey bands, Fig. 4A), indicating preserved haematological homoeostasis despite acute mpox infection. As physiologically expected, males exhibited higher erythroid indices, including haemoglobin (Hgb), red blood cell count (RBC), and haematocrit (HCT), relative to females. At the same time, no sex-based differences were observed in leucocyte subsets or platelet indices.

When stratified by HIV status (Fig. 4B), distinct immunohaematological patterns emerged. Participants without HIV-1 demonstrated significantly higher Hgb (p < 0.001), RBC (p < 0.001), HCT (p < 0.001), and mean corpuscular haemoglobin concentration (MCHC p = 0.011), alongside elevated lymphocytes (p = 0.021) and reticulocyte counts (p = 0.022). These findings are consistent with relatively preserved erythroid indices and higher lymphocyte counts in participants without HIV-1. Conversely, individuals living with HIV-1 exhibited significantly increased red-cell distribution width, as indicated by both the coefficient of variation (RDW-CV and standard deviation (RDW-SD) (both p < 0.001), suggesting pronounced anisocytosis. They also displayed elevated neutrophil counts (p = 0.024) and reduced basophils (p = 0.044), reflecting a modest neutrophilic shift and relative lymphopenia.

No significant differences were observed in total white blood cell counts (WBC), mean corpuscular volume (MCV), mean corpuscular haemoglobin (MCH), monocytes, eosinophils, mean platelet volume (MPV), platelet distribution width (PDW), total platelet count, reticulocyte haemoglobin content, immature granulocytes and nucleated RBCs (all p ≥ 0.063). Collectively, these data indicate that while sex-related differences conformed to expected physiological norms, HIV infection was associated with distinct haematological profile marked by anaemia, anisocytosis, and subtle myeloid skewing.

### Sex-adjusted out-of-range haematology patterns

Using sex-specific reference ranges, 152/155 participants (98.1%) had at least one out-of-range haematological index. Overall, OOR burden was slightly higher in females, whereas erythroid and platelet deviations showed mixed or male-predominant patterns. In contrast, HIV status was the principal driver of variation: PLHIV demonstrated subnormal erythroid indices with elevated RDW, consistent with anaemia and anisocytosis. Detailed distributions and culture-stratified patterns are presented in the Supplementary results (Figure S2A and B).

The white cell differential showed a myeloid-predominant shift, characterised by neutrophilia and relative lymphopenia, while platelet counts and morphology contributed fewer discriminatory OOR events. Taken together with distributional analyses (Fig. 4), these findings demonstrate that, after adjusting for sex-specific reference ranges, HIV is associated with impaired erythropoiesis and a skewed myeloid profile marked by neutrophil predominance and lymphopenia.

### Serum chemistry profiles suggest inflammation with HIV-linked hypoalbuminaemia

**Fig. 5:**
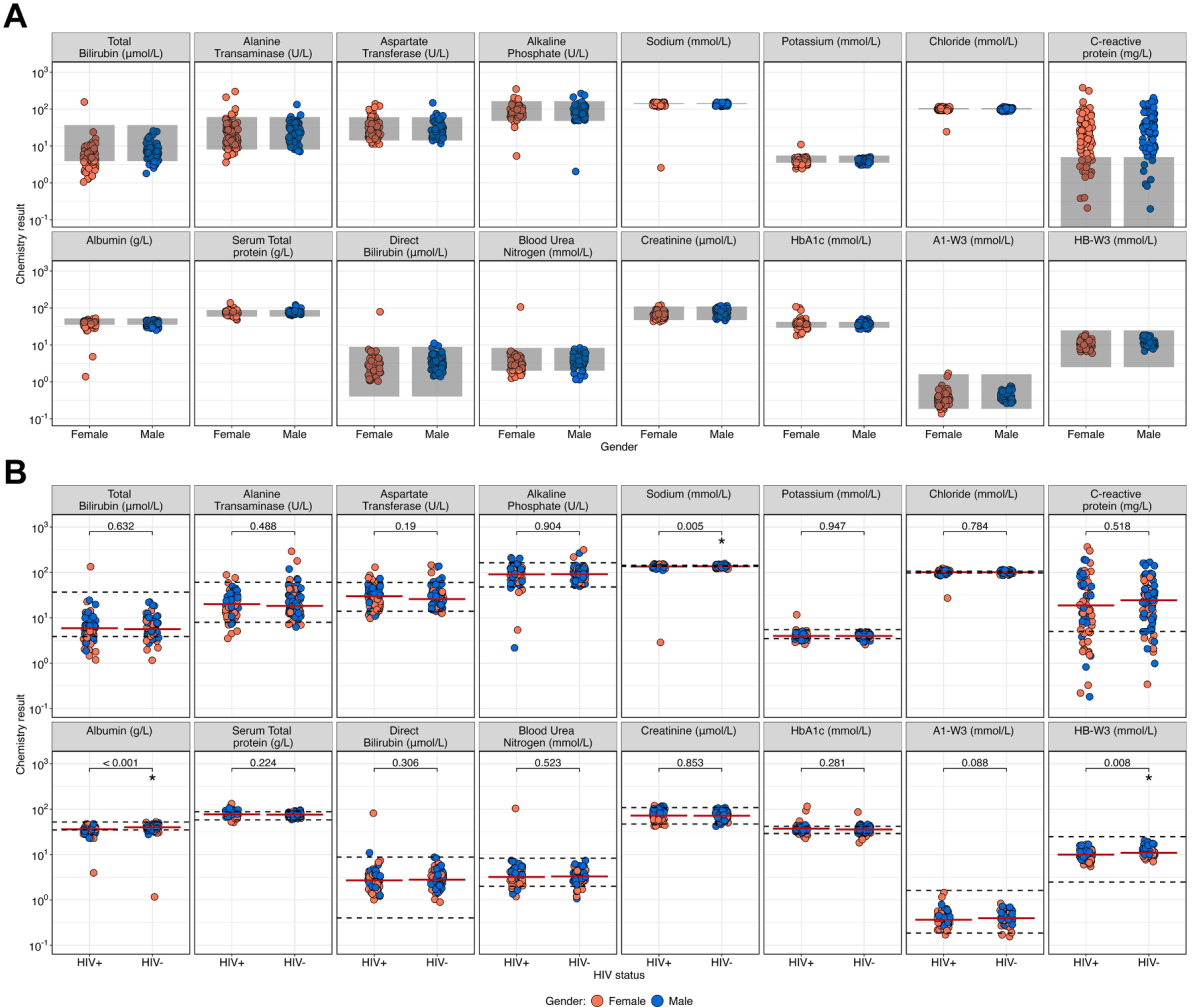
**Serum Chemistry Profiles by Sex and HIV Status**. Individual values for each chemistry analyte are shown. Values are stratified by sex (females, red; male, blue), with grey bands indicating sex-specific reference ranges (**A**). Values are stratified by HIV status (participants with HIV-1 vs prticipants without HIV-1, with points coloured by sex and dashed lines indicating reference limits (**B**). Y axes are displayed on a log_10_ scale where required to accommodate wide dynamic ranges. Two-sided p values for comparisons by HIV status are shown above each panel.

Across the 155-participant cohort, most serum chemistry indices remained within sex-specific reference ranges with minimal sex-related variation (Fig. 5A). Hepatic enzymes, renal markers, electrolytes, HbA1c, and total protein showed substantial overlap between males and females. C-reactive protein displayed the greatest dispersion, consistent with a pervasive inflammatory signal. Stratification by HIV status revealed a focused biochemical imprint (Fig. 5B). Serum albumin was significantly lower in participants living with HIV-1 (p < 0.001), accompanied by a modest reduction in sodium (p = 0.005), while other analytes were broadly comparable between groups (all p ≥ 0.088). Overall, end-organ chemistry remained largely preserved during acute mpox, with HIV-associated hypoalbuminaemia emerging as the principal discriminatory feature. Direction-aware out-of-range analyses are presented in the Supplementary results (Figure S3).

### Lesion viral burden aligns with bacterial co-isolation and inflammation, not HIV status

**Fig. 6:**
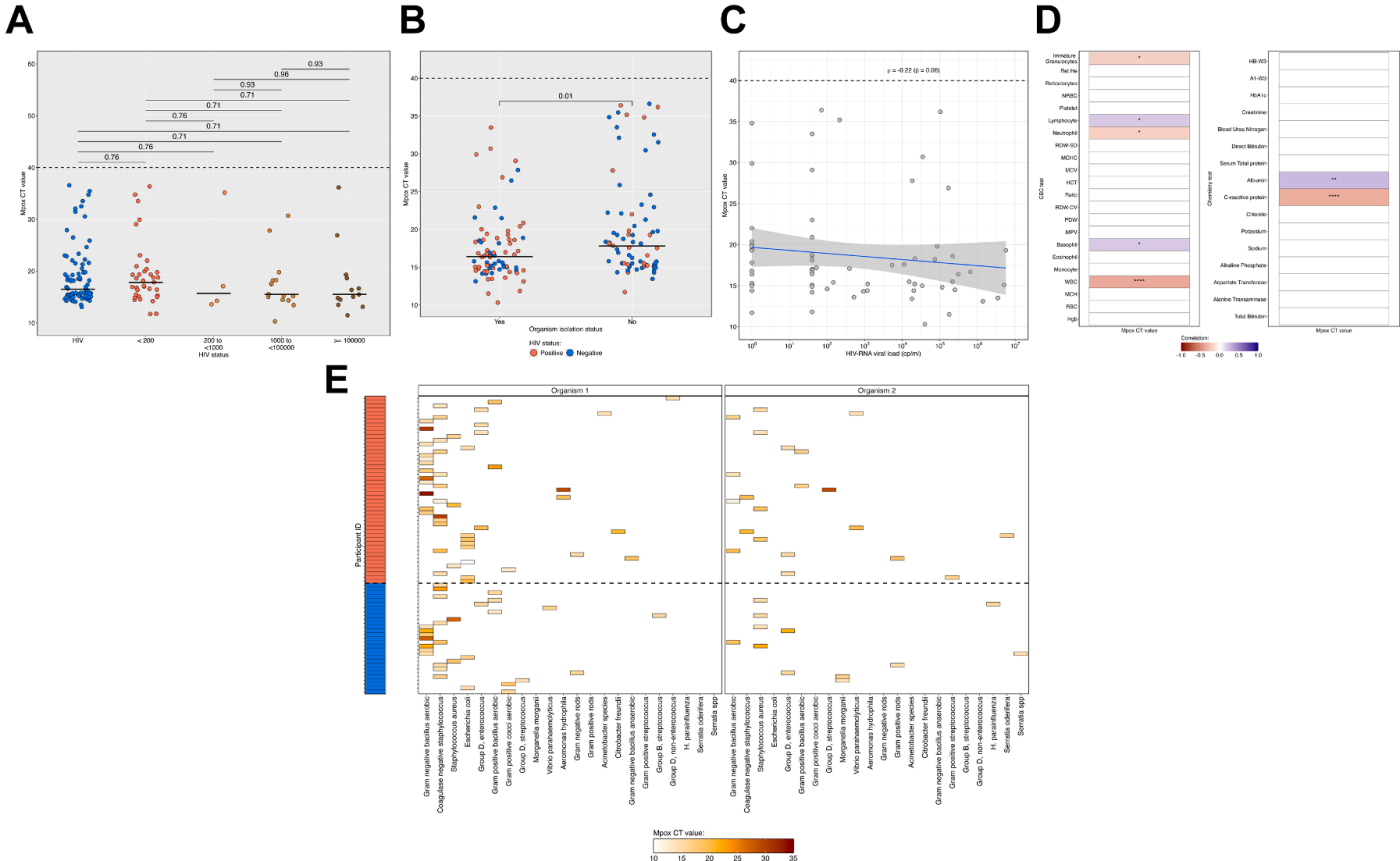
**Lesion Viral DNA burden in relation to HIV status, bacterial co-isolation, and systemic laboratory markers in hospitalised adults**. Lesion viral DNA burden was estimated using qPCR cycle threshold (Ct) values (lower Ct indicates higher viral DNA). Ct values are stratified by HIV serostatus (**A**). Ct values according to bacterial culture status in the analytical dataset (**B**). Relationship between Ct values and plasma HIV RNA among participants with available viral load measurements, with fitted regression and Spearman's correlation (**C**). Spearman correlations between Ct values and selected haematological and serum chemistry indices; only statistically significant associations areannotated (asterisks), and the colour scale indicates direction and magnitude of correlation (**D**). Participant-level distribution of Ct values across recovered bacterial taxa, shown separately for first and second isolates where applicable (**E**). Across panels, Ct values were similar by HIV serostatus, lower in participants who were selected for bacterial culture, weakly associated with plasma HIV-1 RNA, and aligned with a coherent inflammatory haematological and biochemical profiles. Abbreviations: Ct = cycle threshold; HIV = human immunodeficiency virus; PCR = polymerase chain reaction.

Lesion viral DNA burdens estimated by qPCR-derived Ct (lower Ct indicates higher viral DNA) did not differ by HIV serostatus (median = Ct 16–17; p = 0.54), indicating comparable lesion viral burden across HIV strata (Fig. 6A). In contrast, participants with bacterial growth identified in cultured lesions had significantly lower Ct values (p = 0.01), consistent across HIV strata (Fig. 6B). Among PLHIV with available plasma HIV RNA measurements, lesion Ct values showed a weak, non-significant inverse correlation with viraemia (Spearman ρ ≈ −0.22; p = 0.06) (Fig. 6C), suggesting that higher HIV RNA was not strongly associated with lesion viral burden.

Lower Ct values were associated with a distinct inflammatory haematological profile (Fig. 6D), characterised by higher total white blood cell counts, neutrophilia and increased immature granulocytes, consistent with a left-shifted leucocytosis. Ct values correlated positively with lymphocyte and basophils counts, indicating relative lymphocyte depletion at higher viral burden. Serum markers showed concordant directional trends, with lower Ct values associated with higher C-reactive protein and lower albumin.

Participant-level mapping of Ct values across recovered bacterial taxa demonstrated heterogeneous distributions without consistent organism-specific clustering or additive effects in cases with dual isolates (Fig. 6E).

Collectively, lesion Mpox DNA burden was not differentiated by HIV serostatus but aligned with bacterial co-isolation and systemic inflammatory and haematological indices.

### Clade Ib mpox shows limited genomic diversity without clinical or HIV segregation

High-quality whole-genome sequences generated from outbreak specimens clustered unequivocally within Mpox virus Clade I, subclade Ib, with strong bootstrap support. When analysed alongside global and regional reference genomes, Ugandan sequences formed a single, tightly grouped lineage embedded within broader Clade Ib diversity, consistent with a unified transmission network (Figure S4). Branch lengths were short and homogeneous across the maximum-likelihood tree, indicating minimal nucleotide divergence during the study period. No discrete sub-lineages were evident within the outbreak cluster.

Phylogenetic annotation by HIV status and clinical severity demonstrated broad intermixing of people living with HIV-1 and those without across the tree (Figure S5). Participants with severe disease, ICU admission, or fatal outcomes were similarly dispersed across multiple branches, each containing clinically milder cases. No lineage-specific clustering or terminal patterns associated with adverse outcomes were observed. Overall, within-Clade Ib genomic variation did not segregate with HIV status or clinical severity in this cohort.

## Discussion

This prospective inpatient cohort provides an integrated clinical framework linking lesion-level viral burden, lesion microbiology, and routinely available systemic biomarkers in hospitalised adults with Clade Ib mpox in Uganda. By aligning lesion qPCR, targeted bacterial culture, and routine laboratory markers within a single analytic dataset, the study translates outbreak laboratory outputs into clinically actionable signals relevant to inpatient triage and antimicrobial stewardship in resource-constrained settings.

Four practice-relevant observations emerge. First, pain was nearly universal and frequently multifocal, most prominently affecting the anogenital region and the head. This reinforces pain as a defining but underrecognised feature of mpox morbidity. Similar patterns have been reported in outbreaks in Europe and North America, where severe genital and anorectal pain often required opioid analgesia and procedural interventions.^17^^,^^18^^,^^21^^,^^36^^,^^37^ Our findings extend these observations to an African inpatient cohort predominantly infected with Clade Ib.

Second, bacterial co-isolation from purulent lesions was common among individuals sampled for suspected secondary bacterial infection and was frequently polymicrobial. Gram-negative bacilli predominated, and antimicrobial susceptibility testing demonstrated substantial multidrug resistance. Activity of commonly used first-line β-lactams and fluoroquinolones was limited, whereas meropenem retained relatively preserved in-vitro activity. These observations align with emerging reports from Clade Ib outbreaks from Eastern DRC.^15^ However, interpretation of lesion cultures requires caution, as swabs from ruptured or open lesions may capture colonising flora in addition to true pathogens. Nevertheless, against escalating AMR in Africa,^20^^,^^38^, ^39^, ^40^ the high burden of antimicrobial resistance observed in this cohort underscores the need for culture-guided therapy and strengthened antimicrobial stewardship during mpox outbreaks.

Third, the viral burden of the lesion aligned with a coherent inflammatory phenotype. Lower Ct values indicating higher viral DNA levels, were associated with leucocytosis, neutrophilia with left shift, elevated CRP, and hypoalbuminaemia. These findings suggest that lesion viral burden is associated with systemic inflammatory activation during acute mpox. These data complement longitudinal studies describing Mpox viral kinetics and systemic inflammation in other geographical settings.^26^^,^^27^ To our knowledge, this is among the first prospective African cohort to demonstrate harmonised alignment between lesion viral burden and routine haematological and biochemical markers. These patterns were observed despite similar Ct distributions between participants living with HIV-1 and participants without HIV-1, suggesting that lesion viral burden may represent a correlate of acute inflammation in clade Ib mpox.

Fourth, people living with HIV exhibited a distinct haematological profile characterised by lower erythroid indices, increased anisocytosis, and relative myeloid skewing. In contrast, lesion viral DNA burden was comparable by HIV serostatus, and the correlation between lesion Ct values and plasma HIV-1 RNA was weak and not statistically significant. These findings suggest that HIV-related vulnerability may not be fully captured by serostatus alone and may be better contextualised using haematological indices alongside virological assessment. These observations are directionally consistent with reports linking advanced or uncontrolled HIV infection to more severe mpox outcomes.^28^^,^^29^^,^^41^^,^^42^

Although our data do not demonstrate a strong relationship between HIV viraemia and lesion viral burden, incorporation of plasma HIV RNA into inpatient evaluation may still provide clinically relevant context, particularly when interpreted alongside markers of inflammation and haematological perturbation. This interpretation is consistent with reports from other settings, including the global case series by Mitjà et al.,^43^ and others^44^^,^^45^ in which advanced or uncontrolled HIV infection was associated with more severe mpox outcomes. Collectively, these findings support consideration of an integrated approach to clinical assessment that combines HIV status, plasma HIV RNA (where available), and routine laboratory markers, rather than relying on serostatus alone, when evaluating patients admitted with mpox.

At the health-system level, predominance of multidrug-resistant Gram-negative bacteria highlights the intersection between emerging viral epidemics and the growing antimicrobial resistance crisis in Africa,^38^, ^39^, ^40^^,^^46^ including regional reports of high-risk Klebsiella and *E. coli* lineages and rising carbapenemase resistance prevalence.^47^, ^48^, ^49^, ^50^, ^51^ Skin barrier disruption during mpox infection, combined with empiric broad-spectrum antibiotic use, may be associated with conditions that favour the amplification of resistant organisms unless antimicrobial stewardship is integrated into outbreak care pathways.

Several limitations merit consideration. First, the cohort was restricted to hospitalised patients at a single national referral centre and therefore represents individuals with more severe disease; findings may not generalise to outpatient or community-managed Mpox. Second, lesion Ct values are influenced by sampling technique, lesion stage, and anatomical site, and therefore represent a pragmatic rather than absolute measure of viral burden. Third, bacterial cultures from skin lesions cannot fully distinguish between pathogenic infection and colonisation. Fourth, the cross-sectional design limits inference regarding the temporal dynamics of viral burden, bacterial superinfection, and inflammatory responses. Because culture was restricted to purulent lesions with clinical suspicion of superinfection, the observed yield should not be interpreted as the prevalence of bacterial infection across all mpox lesions. Finally, analyses were descriptive and univariable; the cohort size and cross-sectional design precluded multivariable modelling of determinants of viral burden, disease outcomes, or viral evolutionary kinetics.

Despite these limitations, the study demonstrates that integrating lesion qPCR with routine laboratory markers, including complete blood counts, CRP, albumin, and targeted bacterial culture, may provide a practical framework for inpatient risk stratification during mpox outbreaks. Embedding such approaches within national case-management guidelines may help optimise antimicrobial use, identify high-risk patients earlier, and strengthen outbreak preparedness in African health systems facing recurrent emerging viral epidemics.

In conclusion, Clade Ib mpox requiring hospitalisation in Uganda was characterised by high lesion viral burden, substantial multisite pain, systemic inflammation, and frequent Gram-negative multidrug-resistant lesion-associated bacterial co-isolation in those investigated, with additional haematological vulnerability among people living with HIV. Integrating lesion qPCR with CBC indices, CRP, albumin, and targeted culture offers a potentially feasible, low-cost framework for inpatient risk stratification and antimicrobial stewardship in African hospitals. Embedding this approach within national mpox case-management and preparedness systems could reduce morbidity and support judicious antibiotic use.

## Contributors

Jennifer Serwanga (JS), Raymond Ernest Kaweesa (REK), Geoffrey Odoch (GO), Brenda Nairuba (BN), Deborah Mukisa (DM), Rodney Abraham Tumusiime (RAT), Ruth Priscilla Nambi (RPN), Annie Daphine Ntabadde (ADN), Violet Ankunda (VA), Tom Lutalo (TL), Michael Selorm Avumegah (MSA), John Bosco Nsubuga (JBN), Christopher Nsereko (CN), Pontiano Kaleebu (PK), JS conceived and designed the study. REK, GO, BN, JBN, and CN coordinated the clinical cohort and acquired clinical data. GO, DM, RAT, and RPN performed the laboratory investigations, including qPCR, microbiology, antimicrobial susceptibility testing, and sequencing workflows. VA, REK, DM and JS accessed and verified the underlying data. VA conducted the bioinformatic and statistical analyses. JS, REK, PK, and VA interpreted the data. JS and REK drafted the manuscript. All authors critically revised the manuscript, approved the final version, and agreed to be accountable for all aspects of the work.

## Data sharing statement

De-identified individual participant data and the statistical analysis code will be made available upon reasonable request to the corresponding author, subject to approval by the Uganda Virus Research Institute and in accordance with national data-governance regulations and participant consent.

## Declaration of interests

All authors declare no competing interests.

All authors have completed the ICMJE Disclosure Form. No author reports grants or contracts outside the submitted work, royalties or licences, consulting fees, honoraria for lectures, presentations or educational activities, expert testimony payments, support for attending meetings or travel, patents planned, pending or issued, participation on Data Safety Monitoring Boards or Advisory Boards, leadership or fiduciary roles in external organisations, stock ownership or stock options, receipt of equipment, materials, drugs, medical writing support, gifts or other services, or any other financial or non-financial interests that could be perceived to have influenced the work reported in this manuscript.

## Appendix A Supplementary data

Supplementary Figures and Tables

Caption for Supplementary Figures and Tables
