# Supplementary material for: Lesion viral burden, multidrug-resistant superinfection, and HIV-associated haematological vulnerability in hospitalised clade Ib mpox: a prospective cohort study in Uganda
Source: eBioMedicine. 2026 Aug 5;131:106409. doi: 10.1016/j.ebiom.2026.106409 (PMC13471051; doi:10.1016/j.ebiom.2026.106409)
Supplement: Supplementary file 1 [file mmc1.docx]

**SUPPLEMENTARY METHODS**

**Keywords**

mpox; Clade Ib; HIV; antimicrobial resistance; bacterial superinfection; lesion viral burden; hospitalised adults; Uganda

### **Study Design, Setting, and Population**

Between 3 March and 10 April 2025, we conducted a prospective consecutive inpatient cohort study among adults admitted to the mpox isolation unit at Entebbe Regional Referral Hospital (ERRH), Uganda. During this period, ERRH served as the national referral centre for hospitalised mpox care and received patients with severe or complicated disease referred from health facilities across the country. Consecutive enrolment of eligible admissions was used to minimise selection bias within the hospitalised population.

Eligible participants were adults with clinically compatible mpox and visible cutaneous or mucosal lesions with a documented symptom onset date corroborated during enrolment through clinician assessment and paper-based structured epidemiological data capture. At admission, patients were classified as **suspected, probable, or confirmed** mpox according to WHO-aligned case definitions to guide triage and clinical management**; these classifications were not used for analytic stratification in this report.** The **primary analytic cohort comprised adults with laboratory-confirmed mpox**, defined by detection of Mpox virus DNA in lesion swabs by real-time quantitative PCR (qPCR) targeting the F3L gene. Clade Ib assignment was confirmed by whole-genome sequencing in a predefined subset, restricted to specimens with qPCR Ct values ≤15.

Participants were enrolled and sampled at a harmonised acute timepoint of 14 ± 2 days after symptom onset, defined as the first systemic symptom (fever, malaise, or lymphadenopathy), which typically preceded lesion emergence. Specimens collected at this timepoint included saliva, EDTA blood, serum separator tube (SST) blood, and acid citrate dextrose (ACD) blood, together with lesion swabs from skin, genital, and anal sites, where consent was provided. Uptake of lesion sampling varied by anatomical site, with the highest acceptance observed for skin and genital swabs, whereas anal sampling was less frequently accepted.

For diagnostic confirmation and viral burden assessment, lesion swabs constituted the primary specimen type. Where multiple lesion sites were sampled, one lesion-linked qPCR result per participant was selected using a prespecified hierarchy to ensure comparability across participants. Genital lesion swabs were prioritised where available, reflecting the predominance and clinical severity of anogenital disease in this cohort; where genital sampling was unavailable, cutaneous lesion swabs were used. Anal swabs, although collected in a subset, were not routinely prioritised for the primary analysis because of lower uptake. The distribution of specimen types contributing to PCR-confirmed cases is shown in **Supplementary Figure S1**.

Lesion swabs were tested using the BioPerfectus Monkeypox Kit (Jiangsu BioPerfectus Technologies Co., Ltd., China) on a QuantStudio™ 7 Pro real-time PCR platform (Applied Biosystems). All diagnostic and research laboratory investigations were conducted at the Uganda Virus Research Institute (UVRI), Uganda’s national reference laboratory. Purulent lesions meeting predefined clinical criteria were additionally sampled for bacterial culture and antimicrobial susceptibility testing to inform clinical management.

All participants provided written informed consent. The study protocol was approved by the Uganda Virus Research Institute Research and Ethics Committee (UVRI-REC GC/127/1068) and registered with the Uganda National Council for Science and Technology (UNCST; NS934ES).

### **Clinical data collection and case reporting procedures**

Clinical and epidemiological data were collected using clinician-administered structured interviews based on a standardised case report form adapted from the WHO mpox minimum dataset and refined with Ugandan clinician input to reflect local care pathways [29]. Variables collected included age, sex, district of residence, education level, HIV serostatus, pregnancy status where applicable, exposure history, lesion distribution, symptom burden, and clinical severity indicators (**Supplementary Table S1**). Exposure categories were self-reported and non-mutually exclusive and are therefore presented descriptively.

Operational clinical outcomes were defined a priori using data extracted from hospital medical records and structured case report form (CRF) interviews. Clinical severity was assessed using a composite of documented complications, including necrosis, sepsis, and ocular involvement, as recorded by treating clinicians. Intensive care unit (ICU) or high-dependency unit (HDU) admission was defined as documented escalation to higher-level care during hospitalisation. Sepsis was defined based on clinician diagnosis recorded in the medical record in the context of suspected or confirmed infection. Mortality was defined as in-hospital death during the admission episode.

Additional outcomes included length of hospital stay (days from admission to discharge) as a measure of recovery trajectory and healthcare utilisation, and readmission within 14–30 days of discharge, identified through hospital records and follow-up data, to capture early post-discharge outcomes. These definitions were selected to reflect clinically meaningful endpoints in a resource-limited outbreak setting where standardised mpox-specific severity scores are not available

**HIV serostatus Determination**

HIV testing followed the Uganda Ministry of Health HIV Testing Services Policy and Implementation Guidelines (2022), which prescribe a national serial testing algorithm. EDTA plasma from each participant was first screened using the Determine™ HIV-1/2 rapid test (Alere/Abbott, South Korea). Non-reactive samples were classified as HIV-negative. Reactive samples underwent confirmatory testing with STAT-PAK HIV-1/2 (Chembio Diagnostic Systems, USA). Concordant reactivity on both assays was interpreted as HIV-positive. Discordant results were resolved using the SD Bioline HIV-1/2 tiebreaker assay (Standard Diagnostics, South Korea). This nationally standardised serial algorithm ensured consistent classification of HIV serostatus across the cohort.

**Lesion viral DNA burden and HIV-1 RNA quantification**
Mpox lesion viral DNA was quantified from lesion swabs by real-time qPCR. Cycle threshold (Ct) values were used as an inverse proxy for lesion viral burden, with lower Ct values indicating higher viral DNA levels. For the primary Ct analysis, one lesion-linked qPCR result per participant was selected using a prespecified hierarchy, with genital swabs prioritised where available and alternative lesion-site swabs used otherwise. Ct distributions were compared by HIV serostatus, lesion anatomical site, bacterial growth identified in cultured lesions, and clinical indicators of suspected secondary infection.

Among people living with HIV, plasma HIV-1 RNA was quantified using the Xpert HIV-1 Viral Load XC version 3 nucleic acid amplification assay on the GeneXpert System (software version R2-5.1; lower limit of detection 5 copies/mL). Results were reported as copies per mL and analysed both continuously and using programmatically relevant categories: suppressed (<200 copies/mL), low-level viraemia (200 to <1000 copies/mL), unsuppressed viraemia (1000 to <100000 copies/mL), and very high-level viraemia (≥100000 copies/mL). Antiretroviral therapy exposure was self-reported; viral suppression status was therefore defined objectively using plasma HIV-1 RNA.

### **Haematology and serum biochemistry**

Peripheral venous blood was collected into EDTA tubes and analysed using a Mindray BC-6200 automated haematology analyser according to institutional standard operating procedures. Complete blood count parameters included erythroid indices (haemoglobin, haematocrit, mean corpuscular volume, mean corpuscular haemoglobin, mean corpuscular haemoglobin concentration, red-cell distribution width), white blood cell differentials (neutrophils, lymphocytes, monocytes, eosinophils, basophils, immature granulocytes), platelet indices, and reticulocyte measurements.

Serum biochemistry included hepatic markers (alanine aminotransferase [ALT], aspartate aminotransferase [AST], albumin, and total protein), renal markers (creatinine, urea), and inflammatory markers (C-reactive protein [CRP]). Results were interpreted using sex-specific reference ranges and classified as within or outside reference limits.

### **Bacterial culture and antimicrobial susceptibility testing**

Purulent lesions were sampled using sterile swabs from cutaneous or mucosal pustular lesions when clinically evident. Specimens were transported and processed on the day of collection. Aerobic bacterial culture was performed using standard microbiological media. Isolates were identified to species level using conventional biochemical testing supplemented by automated identification systems.

Antimicrobial susceptibility testing was conducted using EUCAST-standardised disk diffusion methods. Inhibition zone diameters were interpreted according to EUCAST clinical breakpoints as susceptible, intermediate, or resistant. The antibiotic panel included β-lactams (ampicillin, benzylpenicillin, amoxicillin-clavulanic acid, ceftriaxone), ciprofloxacin, clindamycin, co-trimoxazole, erythromycin, gentamicin, meropenem, and vancomycin.

Quality assurance included reference strains (*Escherichia coli* ATCC 25922, *Staphylococcus aureus* ATCC 25923, *Pseudomonas aeruginosa* ATCC 27853, *Enterococcus faecalis* ATCC 29212). Ambiguous results or out-of-control runs triggered repeat testing. Culture results were interpreted as lesion-associated bacterial co-isolates recovered from purulent lesions.

### **Whole-genome sequencing and phylogenetic analysis**

Whole-genome sequencing was performed on a subset of specimens (n= 40) with sufficient viral DNA (Ct ≤15). Viral DNA was extracted using the QIAamp Viral DNA Mini Kit (QIAGEN). Amplicon-based libraries spanning the approximately 190 kb Mpox virus genome were generated using an overlapping primer set, as previously described (30).

Libraries were sequenced on an Illumina MiSeq platform. Raw reads were processed using both the Illumina DRAGEN Microbial Amplicon Pipeline and a validated in-house workflow. Consensus genomes were compared across pipelines for concordance. Consensus genomes were assigned clades using Nextclade (v3.18.0) and phylogenetically placed within curated global mpox reference datasets. Only genomes with >95% genome coverage and <1% ambiguous bases were retained for downstream phylogenetic analyses.

**Statistical Analysis**

All analyses were performed in R (v4.4.1). Categorical variables are summarised as counts and proportions, and continuous variables as medians with interquartile ranges (IQRs). Primary analyses evaluated associations between HIV serostatus, lesion viral DNA burden, bacterial growth from cultured purulent lesions, and antimicrobial susceptibility patterns among hospitalised adults with mpox.

Group differences in haematological and biochemical parameters by HIV serostatus were assessed using the Wilcoxon rank-sum test. Associations between HIV serostatus and bacterial growth, and between HIV serostatus and susceptibility to individual antibiotics, were assessed using Fisher’s exact test due to small cell counts; effect sizes are presented as odds ratios with 95% confidence intervals. Correlations between quantitative variables, including lesion qPCR cycle threshold (Ct) values, plasma HIV-1 RNA among people living with HIV, and selected laboratory parameters, were assessed using Spearman’s rank correlation coefficient.

Heatmaps were used to visualise participant-level heterogeneity in lesion viral burden, PCR positivity across sampled anatomical compartments, and antimicrobial susceptibility profiles. All statistical tests were two-sided, with p<0.05 considered statistically significant.

Exploratory analyses of haematological and biochemical parameters between groups (e.g., HIV-positive vs HIV-negative; male vs female) were conducted separately for each laboratory analyte, with each test representing a distinct biological outcome. These analyses were therefore treated as independent evaluations rather than repeated testing of a single outcome, and no formal adjustment for multiple comparisons was applied.

No a priori power calculation was performed because the cohort comprised consecutive inpatient admissions during the outbreak period. Missing data were not imputed; analyses were conducted on available observations, with variable-specific denominators reported where applicable.

**SUPPLEMENTARY RESULTS**

**Cohort derivation and exposure reporting**

A total of 157 inpatients with clinically compatible mpox were enrolled during the study period. Two paediatric cases (aged 7 and 9 years) were excluded from adult analyses, leaving a primary analytic cohort of 155 adults with PCR-confirmed mpox. Clade Ib assignment was confirmed by whole-genome sequencing in a predefined subset, , restricted to specimens with qPCR Ct values ≤15.

Structured case-report interviews were available for 146/155 participants (94.2%) and captured demographic characteristics, pregnancy status, exposure history, and lesion distribution. Admission triage classifications (suspected, probable and confirmed) reflected the clinical care pathway at presentation and are shown in **Supplementary Table S1** for completeness, but were not used for analytic stratification. All included participants had laboratory-confirmed infection. Exposure categories were self-reported and non-mutually exclusive; proportions should therefore be interpreted descriptively rather than as attributable transmission risks.

**Exploratory out-of-range haematology analysis**

Using sex-specific reference ranges [31, 32], CBC profiling showed that 152 of 155 participants (98.1%) had ≥1 out-of-range (OOR) parameter (**Supplementary Figure S2**). Total OOR burden was modestly higher among females. However, selected erythroid and platelet indices (MCH, MPV, PDW, HCT, MCV, MCHC, reticulocytes) were more frequently elevated in males or showed no consistent sex pattern (**Supplementary** **Figure S2A**).

Among participants with culture-confirmed bacterial infection, the OOR burden was broader, with MPV, PDW, HCT, MCHC, and reticulocyte deviations more often exceeding upper reference thresholds, consistent with anaemia and anisocytosis .

Compared with sex, HIV serostatus showed a more consistent pattern of haematological disturbance. Participants living with HIV exhibited consistently reduced erythroid indices (Hgb, RBC, HCT) alongside increased RDW (CV and SD), consistent with anaemia and anisocytosis (**Supplementary Figure S2B**).

**Exploratory out-of-range serum biochemistry analysis**

Direction-aware analysis of out-of-range (OOR) serum biochemistry confirmed CRP as the dominant abnormality across the cohort, with elevations above the upper reference limit occurring most frequently (**Supplementary** **Figure S3**). This pattern was observed in both sexes across HIV strata, supporting a broad inflammatory profile during acute hospitalised mpox.

In contrast, reductions below the reference range were most evident for albumin, sodium, potassium, and total bilirubin, occurring disproportionately among people living with HIV and, to a lesser extent, females. Chloride deviations were predominantly upward, whereas ALT, AST, and alkaline phosphatase (ALP) abnormalities were uncommon. Minimal OOR burden was observed for creatinine, BUN, total protein, direct bilirubin, and HbA1c. Together, these secondary analyses reinforce the main finding that systemic inflammation is widespread in acute mpox, while HIV infection preferentially associates with hypoalbuminaemia and mild electrolyte perturbation.

**Supplementary Figure S1**: Study cohort derivation, data availability, and analytical subsets in the hospitalised Clade Ib mpox cohort.

Flow diagram showing participant inclusion, data availability, and key analytical subsets. Of 157 enrolled participants, two paediatric cases were excluded, leaving 155 adults in the primary analytical cohort. Among these, 81 were female, 74 were male, 73 were living with HIV-1 and 82 were HIV-negative. Demographic and exposure data were available for 146 participants. Within this subgroup, admission classification was recorded as confirmed in 32, suspected in 107, probable in five, and unclassified in two. Complete blood count, serum biochemistry, and primary lesion qPCR Ct data were available for all 155 adults, 80 underwent bacterial culture of purulent lesions. Abbreviations: Ct, cycle threshold; HIV, human immunodeficiency virus; PCR, polymerase chain reaction. “Isolation” refers to participants sampled for bacterial culture.

**Supplementary Figure S2:** Distribution of **out-of-range complete blood count parameters by sex and HIV serostatus (n=155).**

A

B

**Figure S2A** shows the number of participants with complete blood count values outside sex-specific reference ranges, stratified by sex. **Figure S2B** shows the same parameters stratified by HIV serostatus. Stacked bars indicate the direction of deviation from the reference range, with blue denoting values above the upper limit and orange values below the lower limit. Axes and scales are parameter-specific.

**Supplementary Figure S3:** Distribution of out-of-range serum biochemistry parameters by sex and HIV serostatus (n=155)

**Figure S3A** shows the number of participants with serum biochemistry values outside reference ranges, stratified by sex. **Figure S3B** shows the same parameters stratified by HIV serostatus. Stacked bars indicate the direction of deviation from the reference range, with blue representing values above the upper reference limit and orange values below the lower reference limit. Reference limits are sex-specific where applicable; axes and scales are analyte-specific.

**Supplementary Figure S4:** **Phylogenetic placement of Mpox virus genomes including Ugandan sequences.**

Clade Ib

Clade IIa

Clade Ia

Clade IIb

**Supplementary Figure S4 shows the** maximum-likelihood phylogeny of Mpox virus genomes coloured by lineage or reference group. Ugandan sequences generated in this study are highlighted in pink. Branch lengths represent genetic distance, and reference genomes from other clades are included for context. Tips correspond to individual genome accessions.

**Supplementary Figure S5: Maximum-likelihood cladogram of Ugandan Mpox virus genomes annotated by HIV serostatus and clinical severity (n = 40).**

Maximum likelihood phylogeny showing relationships among 40 Ugandan Mpox virus genomes with **PP601219.1** included as a reference sequence. Tip labels correspond to individual study samples and are coloured by HIV serostatus (blue, HIV-negative; magenta, HIV-positive). Clinical severity annotations include **severe disease (n = 2)**, **ICU admission (n = 1)**, and **fatal outcomes (n = 2)**. Branch lengths represent genetic distance, and bootstrap values indicate node support.

**Supplementary Table S1:** Baseline characteristics of interviewed adult inpatients with laboratory-confirmed mpox, stratified by WHO admission triage classification (n = 144).

| **Characteristic** | | Total  (Col %) | Confirmed  (Col %) | Suspected  (Col %) | Probable  (Col %) |
| --- | --- | --- | --- | --- | --- |
| **Total** | | 144 (100) | 32 (22.2) | 107 (74.3) | 5 (3.5) |
| **Age** (median/IQR) | | 30.0 (25.0-36.0) | 30.0 (25.0-35.0) | 30.0 (25.0-36.0) | 25.0 (25.0-35.0) |
| **Sex** | |  |  |  |  |
|  | Female | 72 (50.0) | 13 (40.6) | 56 (52.3) | 3 (60.0) |
|  | Male | 72 (50.0) | 19 (59.4) | 51 (47.7) | 2 (40.0) |
| **District of residence** | |  |  |  |  |
|  | Kampala | 81 (56.2) | 18 (56.2) | 60 (56.1) | 3 (60.0) |
|  | Wakiso | 47 (32.6) | 10 (31.2) | 36 (33.6) | 1 (20.0) |
|  | Other districts | 16 (11.1) | 4 (12.5) | 11 (10.3) | 1 (20.0) |
| **Level of Education** | |  |  |  |  |
|  | No formal Education | 9 (6.2) | 2 (6.2) | 7 (6.5) | 0 (0.0) |
|  | Primary | 53 (36.8) | 9 (28.1) | 43 (40.2) | 1 (20.0) |
|  | Secondary | 57 (39.6) | 20 (62.5) | 37 (34.6) | 0 (0.0) |
|  | Tertiary/University | 5 (3.5) | 0 (0.0) | 5 (4.7) | 0 (0.0) |
|  | No Information | 20 (13.9) | 1 (3.1) | 15 (14.0) | 4 (80.0) |
| **ICU Admission** | |  |  |  |  |
|  | Yes | 4 (2.8) | 4 (12.5) | 0 (0.0) | 0 (0.0) |
|  | No | 133 (92.4) | 26 (81.2) | 102 (95.3) | 5 (100.0) |
|  | No information | 7 (4.9) | 2 (6.2) | 5 (4.7) | 0 (0.0) |
| **STI in the last year** | |  |  |  |  |
|  | Yes | 37 (25.7) | 3 (9.4) | 32 (29.9) | 2 (40.0) |
|  | No | 77 (53.5) | 16 (50.0) | 58 (54.2) | 3 (60.0) |
|  | No Information | 30 (20.8) | 13 (40.6) | 17 (15.9) | 0 (0.0) |
| **HIV serostatus** | |  |  |  |  |
|  | Positive | 69 (47.9) | 17 (53.1) | 51 (47.7) | 1 (20.0) |
|  | Negative | 75 (52.1) | 15 (46.9) | 56 (52.3) | 4 (80.0) |
| **Previous mpox diagnosis** | |  |  |  |  |
|  | Yes | 2 (1.4) | 1 (3.1) | 0 (0.0) | 1 (20.0) |
|  | No | 131 (91.0) | 30 (93.8) | 97 (90.7) | 4 (80.0) |
|  | No Information | 11 (7.6) | 1 (3.1) | 10 (9.3) | 0 (0.0) |

Baseline characteristics of interviewed adult inpatients with laboratory-confirmed mpox, stratified by admission triage classification within the routine clinical care pathway. The table summarises demographic and clinical characteristics for the overall interviewed cohort with classified admission status (n=144) and for participants classified at admission as confirmed (n=32), suspected (n=107), or probable (n=5). Variables are presented as n (column %) unless otherwise indicated; age is summarised as median (IQR). Percentages are calculated within each admission classification column. The table (n=144) excludes two interviewed participants with unclassified admission status.

**Supplementary Table S2.** Distribution of antimicrobial susceptibility interpretations across tested organism-antibiotic combinations from purulent mpox lesions.

| **Antibiotic (total tests)** | **Antibiotic interpretation** | **Count (Percentage)** |
| --- | --- | --- |
| Amoxicillin-Clavulanic acid  (Augmentin) (n = 49) | Resistant | 44 (89.8%) |
|  | Susceptible | 5 (10.2%) |
| Ampicillin (n = 2) | Resistant | 2 (100%) |
|  | Susceptible | 0 (0.0) |
| Benzylpenicillin (n = 30) | Resistant | 29 (96.7%) |
|  | Susceptible | 1 (3.3%) |
| Ceftriaxone (n = 38) | Resistant | 30 (78.9%) |
|  | Susceptible | 8 (21.1%) |
| Ciprofloxacin (n = 111) | Resistant | 90 (81.1%) |
|  | Susceptible | 21 (18.9%) |
| Clindamycin (n = 32) | Resistant | 18 (56.2%) |
|  | Susceptible | 14 (43.8%) |
| Co-trimoxazole (n = 47) | Resistant | 32 (68.1%) |
|  | Susceptible | 15 (31.9%) |
| Erythromycin (n = 24) | Resistant | 18 (75.0%) |
|  | Susceptible | 6 (25.0%) |
| Gentamicin (n = 87) | Resistant | 45 (51.7%) |
|  | Susceptible | 42 (48.3%) |
| Meropenem (n = 61) | Resistant | 10 (16.4%) |
|  | Susceptible | 51 (83.6%) |
| Penicillin (n = 5) | Resistant | 5 (100%) |
|  | Susceptible | 0 (0.0%) |
| Vancomycin (n = 17) | Resistant | 10 (58.8%) |
|  | Susceptible | 7 (41.2%) |

Counts and percentages represent organism-antibiotic test combinations interpreted for each antibiotic. Percentages are calculated using the total number of isolates tested against that specific antibiotic; denominators therefore vary by antibiotic because not all isolates were tested against all drugs. Results describe the distribution of susceptibility interpretations across tested combinations and should not be interpreted as participant-level prevalence estimates. Intermediate calls were collapsed to susceptible

**Graphical Abstract**
