# Supplementary material for: Lesion viral burden, multidrug-resistant superinfection, and HIV-associated haematological vulnerability in hospitalised clade Ib mpox: a prospective cohort study in Uganda
Source: eBioMedicine. 2026 Aug 5;131:106409. doi: 10.1016/j.ebiom.2026.106409 (PMC13471051; doi:10.1016/j.ebiom.2026.106409)
Supplement: Supplementary file 2 [file mmc2.docx]

**Caption for Supplementary Material**

**Supplementary Methods.**

Detailed description of study design, participant recruitment, specimen collection, laboratory procedures, bacterial culture and antimicrobial susceptibility testing, whole-genome sequencing, and statistical analysis framework for the hospitalised Clade Ib mpox cohort.

**Supplementary Results.**

Expanded results describing cohort derivation, exposure reporting, exploratory analyses of out-of-range haematological and biochemical parameters, and additional subgroup findings.

**Supplementary Figure S1.**

Study cohort derivation, data availability, and analytical subsets in the hospitalised Clade Ib mpox cohort. Flow diagram illustrating participant inclusion, exclusions, and availability of key datasets, including PCR confirmation, bacterial culture, and clinical metadata.

**Supplementary Figure S2.**

Distribution of out-of-range complete blood count parameters by sex and HIV serostatus (n = 155). Stacked bar plots showing direction and frequency of deviations from sex-specific reference ranges.

**Supplementary Figure S3.**

Distribution of out-of-range serum biochemistry parameters by sex and HIV serostatus (n = 155). Visualisation of analyte-specific deviations above and below reference limits across study subgroups.

**Supplementary Figure S4.**

Phylogenetic placement of Mpox virus genomes including Ugandan sequences. Maximum-likelihood phylogeny showing genetic relationships and clade context of sequenced isolates.

**Supplementary Figure S5.**

Maximum-likelihood cladogram of Ugandan Mpox virus genomes annotated by HIV serostatus and clinical severity (n = 40). Phylogenetic relationships with overlay of clinical metadata, including severe disease and mortality.

**Supplementary Table S1.**

Baseline characteristics of interviewed adult inpatients with laboratory-confirmed mpox, stratified by WHO admission triage classification (n = 144).

**Supplementary Table S2.**

Distribution of antimicrobial susceptibility interpretations across organism–antibiotic combinations from purulent mpox lesions, with antibiotic-specific denominators.

**Supplementary Table S3.**

Data points excluded from primary analyses due to exceeding predefined thresholds.

**Supplementary Data File 1.**

Underlying datasets supporting the main and supplementary analyses, including laboratory measurements, clinical variables, and antimicrobial susceptibility data.
